# Improving Awareness and Accessibility of Well-being Resources Among Core Psychiatric Trainees in the West Midlands Deanery- a Quality Improvement Project

**DOI:** 10.1192/bjo.2022.272

**Published:** 2022-06-20

**Authors:** Nneamaka Asiodu, Ellen Williams, Amitav Narula

**Affiliations:** Black Country Healthcare NHS Foundation Trust, Walsall, United Kingdom

## Abstract

**Aims:**

Supporting the mental health and well-being of psychiatrists impacts on the quality of patient care delivered, and crucially for trainees, on retention to the profession. Our aim was to survey core trainees to gauge their awareness, access and use of well-being resources.

**Methods:**

111 core trainees in the West Midlands deanery were invited to complete an anonymous online survey during November 2020. Quantitative data were analysed using Google Forms and Microsoft Excel. Qualitative data were reviewed by all team members to identify relevant themes.

**Results:**

Only 14% of trainees felt well informed about the well-being resources available to them, 57% who attended local trust induction and 82% who attended deanery induction did not think nor recall if the topic had been covered. Despite this, trainees were aware of a range of resources, with the most known being BMA Wellbeing (58.3%), Psychiatrists’ Support Service (44.4%) and the local Peer Support Unit (30.6%). Just 14% of trainees reported using a well-being resource during their training.

**Conclusion:**

Our results suggest that more work needs to be done at local trust and deanery level to make well-being a priority during induction and improve awareness of available resources. At least one trainee reported they would not seek help, due to concerns about the impact on their training progression, and stigma may be an under-reported barrier to accessing these resources.

We hope to use our results to improve signposting, and to collate a resource easily accessible and applicable to all trainees in the deanery.

